# Dynamic changes in aqueous humor cytokines and OCTA parameters during aflibercept loading in treatment-naive neovascular age-related macular degeneration

**DOI:** 10.3389/fmed.2026.1857826

**Published:** 2026-07-30

**Authors:** Chenwei Gui, Xinxia Chen, Haitao Liu, Jialong Dong, Qing Zhang, Yan Gao, Qing Peng

**Affiliations:** 1 Department of Ophthalmology, Shanghai Tenth People’s Hospital, Tongji University School of Medicine, Shanghai, China; 2 Department of Ophthalmology, Shanxi Eye Hospital Affiliated to Shanxi Medical University, Taiyuan, Shanxi, China

**Keywords:** aflibercept, aqueous humor biomarkers, neovascular age-related macular degeneration, optical coherence tomography angiography, retinal vessel density

## Abstract

**Purpose:**

To investigate dynamic changes in aqueous humor cytokines and retinal vessel density during aflibercept loading in treatment-naive neovascular age-related macular degeneration (nAMD), and to analyze their associations with visual improvement.

**Methods:**

This single-center retrospective clinical study included 20 treatment-naive patients with nAMD (20 eyes) and 20 patients with cataracts (20 eyes). In the nAMD group, aqueous humor samples were collected at baseline and at the time of the third intravitreal aflibercept injection, while samples from the cataract control group were obtained before surgery. Relevant aqueous humor cytokines were measured using the Bio-Plex suspension bead array system. In the nAMD group, best-corrected visual acuity (BCVA) and optical coherence tomography angiography (OCTA) examinations were performed at baseline and at 1 and 2 months after treatment. Longitudinal changes in OCTA parameters were analyzed using mixed-effects models, followed by correlation analyses. According to the final BCVA outcome, eyes in the nAMD group were further classified into an obvious-improvement group and a non-obvious-improvement group for exploratory subgroup comparison.

**Results:**

Compared with the cataract control group, eyes with nAMD showed significant differences in baseline aqueous humor levels of Ang-2, HGF, VEGF-C, IP-10, HB-EGF, IL-8, and PlGF. During aflibercept loading, aqueous humor levels of Ang-2, HGF, and IP-10 increased, whereas IL-8 decreased. At month 2, the superficial parafoveal vessel density and the superficial and deep perifoveal vessel density decreased significantly compared with baseline, while BCVA improved significantly. Baseline Ang-2, bFGF, and IL-8 showed nominal exploratory associations with OCTA changes, but these associations did not remain significant after FDR correction. In subgroup analysis of the nAMD cohort, only the change in deep perifoveal vessel density differed significantly between the obvious-improvement group and the non-obvious-improvement group.

**Conclusion:**

Treatment-naive nAMD eyes exhibited differential changes in aqueous humor cytokines during aflibercept loading, consistent with possible non-VEGF pathway involvement. Meanwhile, regional remodeling of retinal microcirculation was observed, and changes in deep perifoveal vessel density showed a trend consistent with short-term visual improvement.

## 1 Introduction

Age-related macular degeneration is a major cause of central vision impairment and blindness in older adults. Among its subtypes, neovascular age-related macular degeneration (nAMD) accounts for a smaller proportion of cases but remains the principal form responsible for severe visual loss, and its disease burden is expected to continue to rise with population aging (1). Intravitreal anti-vascular endothelial growth factor (VEGF) therapy remains the standard first-line treatment for nAMD, yet the need for repeated injections, variable durability, and heterogeneous anatomical and functional responses continue to limit long-term management (2, 3).

Current evidence suggests that nAMD is not driven by VEGF alone (4–6). In addition to VEGF-dependent angiogenesis and vascular leakage, vascular destabilization, chronic low-grade inflammation, and alternative pro-angiogenic pathways are also involved (7). Among these, angiopoietin-2 (Ang-2) has attracted particular attention because of its links to endothelial activation, leakage, and inflammatory amplification (8, 9), and the clinical development of dual-pathway agents such as faricimab further supports the relevance of VEGF-independent signaling in nAMD (10, 11). Accordingly, identifying intraocular biomarkers that reflect disease activity and treatment-response heterogeneity has become an important goal.

Against this background, the identification of intraocular biomarkers that reflect disease activity, activation of alternative pathways, and variability in treatment response has become an important research priority in nAMD. Aqueous humor is a relatively accessible ocular fluid and has been widely used to profile angiogenic and inflammatory mediators in nAMD (5, 12, 13). Previous studies have shown that cytokines such as IP-10, IL-6, and IL-8 may be abnormal at baseline and may not decline uniformly after anti-VEGF therapy (14–16). In parallel, optical coherence tomography angiography (OCTA) can separately visualize the superficial and deep vessel density, thereby providing a noninvasive quantitative approach for assessing microcirculatory changes in the macular region in nAMD (17). Recent studies have emphasized that OCTA may help identify the morphology and activity of neovascular lesions and may offer information beyond conventional structural parameters for treatment monitoring and prognostic assessment (18, 19). However, it remains unclear which OCTA-derived regional or layer-specific parameters most closely reflect early treatment response and short-term visual benefit.

Most previous studies have evaluated aqueous humor biomarkers, imaging biomarkers, or treatment efficacy separately. Integrated evidence linking cytokine remodeling, OCTA vascular changes, and visual outcomes during the aflibercept loading phase is still limited (20–22). Therefore, this study enrolled treatment-naive eyes with nAMD and cataract control eyes, quantified multiple aqueous humor cytokines, and assessed best-corrected visual acuity and layer-specific OCTA vessel density at baseline, 1 month, and 2 months. The aim of this exploratory study was to examine the associations among intraocular cytokine profiles, regional retinal microvascular remodeling, and short-term visual outcomes in nAMD, and to provide preliminary evidence regarding selective intraocular pathway remodeling during aflibercept loading treatment.

## 2 Materials and methods

### 2.1 Study design and participants

This single-center retrospective observational study included 20 patients (20 eyes) with treatment-naive nAMD treated at the Department of Ophthalmology, Shanghai Tenth People’s Hospital. In parallel, 20 patients (20 eyes) undergoing routine phacoemulsification for age-related cataract during the same period were enrolled as controls. The study was approved by the Institutional Review Board of Shanghai Tenth People’s Hospital (ChiCTR2100051795) and was conducted in accordance with the Declaration of Helsinki. Written informed consent was obtained from all participants.

Eligible patients in the nAMD group were 50 to 90 years of age, had active choroidal neovascularization involving or adjacent to the fovea, had not received prior or recent ocular treatment that could interfere with study evaluation, and had available aqueous humor samples and OCTA follow-up data for the main analyses. Exclusion criteria included substantial scar formation, fibrosis, or subfoveal atrophy in the study eye; active ocular infection; choroidal neovascularization secondary to causes other than AMD; other ocular diseases affecting macular assessment or central vision; any previous anti-VEGF therapy, photodynamic therapy, retinal laser treatment, corticosteroid exposure, or related interventions within the previous 3 months; and systemic autoimmune disease, significant bleeding tendency, severe infection, or other uncontrolled systemic conditions.

The cataract control group consisted of patients undergoing routine cataract surgery during the same period and without other clinically relevant ocular disease. Patients were excluded if they had other ocular disorders affecting macular structure or visual function, recent vitreous hemorrhage, active infection, autoimmune disease, significant bleeding tendency, or severe uncontrolled systemic disease.

### 2.2 Ophthalmic examinations and OCTA acquisition

Patients in the nAMD group underwent ophthalmic examinations at baseline, 1 month, and 2 months, whereas cataract controls underwent routine preoperative ophthalmic assessment. Examinations included best-corrected visual acuity (BCVA), intraocular pressure measurement, slit-lamp examination, and fundus evaluation. BCVA was measured using a standard visual acuity chart and converted to logarithm of the minimum angle of resolution (logMAR) units for statistical analysis.

In the nAMD group, OCTA was performed using the Optovue system (Optovue Inc., USA) with a 6 × 6 mm macular scan protocol. To minimize interference from shadowing and artifacts caused by overlying superficial retinal vessels, a validated three-dimensional projection artifact removal algorithm provided by the manufacturer was applied to all scans. Retinal vessel density in the superficial and deep retinal layers was recorded. The macular region was subdivided according to the built-in OCTA analysis grid as follows: fovea, the central 1-mm-diameter area; parafovea, the annulus extending from 1 to 3 mm in diameter; and perifovea, the annulus extending from 3 to 6 mm in diameter. Only images with a scan quality score >6/10 were included. To ensure the reliability of the quantitative data, all automated segmentation results were manually reviewed and, if necessary, independently corrected by two experienced retinal specialists who were masked to the clinical data. Any discrepancies were resolved by consensus or adjudicated by a third senior specialist to minimize segmentation error.

### 2.3 Treatment protocol and aqueous humor collection

All intravitreal aflibercept injections were performed by the same experienced retinal specialist. Patients in the nAMD group received an aflibercept loading regimen consisting of three monthly injections. Aqueous humor samples were collected immediately before the first injection and again before the third injection by anterior chamber paracentesis at the limbus under sterile conditions using a 30-gauge needle. These two time points were selected to represent the pretreatment baseline and the accumulated early treatment status during the loading phase, while avoiding additional invasive aqueous sampling during routine care. Approximately 100 μL of aqueous humor was obtained at each time point. In the cataract control group, an equal volume of aqueous humor was collected immediately before cataract surgery using the same procedure. All samples were transferred immediately into prelabeled sterile microcentrifuge tubes and stored at −80°C until analysis.

### 2.4 Aqueous humor cytokine measurement

Aqueous humor cytokines were measured using the Bio-Plex 200 suspension bead array system (Bio-Rad, USA), with assay reagents obtained from the same manufacturer. The analytes included Ang-2, HGF, PDGF-AA, VEGF-C, IP-10, bFGF, HB-EGF, IL-8, and PlGF. All samples were processed and analyzed according to a standardized protocol to ensure inter-sample comparability.

### 2.5 Statistical analysis

Continuous variables were summarized as mean ± standard deviation or median (interquartile range [IQR]), as appropriate according to data distribution, whereas categorical variables were expressed as counts and percentages. Baseline comparisons between the nAMD and cataract groups were performed using the independent-samples *t*-test or the Mann–Whitney U test as appropriate, and categorical variables were compared using Fisher’s exact test.

Paired comparisons of aqueous humor cytokines and BCVA before and after treatment in the nAMD group were performed using the paired t test or the Wilcoxon signed-rank test according to data distribution. Longitudinal changes in OCTA parameters were evaluated using a mixed-effects model, followed when necessary by *post hoc* multiple-comparison correction with baseline as the reference. Correlation analyses were performed using Spearman rank correlation. Because the change in deep perifoveal vessel density was the only OCTA parameter that differed significantly between eyes with and without meaningful visual improvement, the additional subretinal fluid (SRF)-based analysis was restricted to this parameter. SRF resolution was defined as complete absence of SRF on structural OCT at month 2. Changes in deep perifoveal vessel density were compared between eyes with and without SRF resolution using the Mann–Whitney U test. All tests were two-sided, and a *P* value < 0.05 was considered statistically significant. Cytokine–OCTA correlations were adjusted using the Benjamini–Hochberg false discovery rate (BH-FDR) procedure. Sensitivity analyses for responder status used 0.05– and 0.15-logMAR thresholds. OCTA mixed-effects analyses were performed in GraphPad Prism 9.5.1, with time as the fixed effect and subject/eye as the repeated-measures unit; Dunnett-adjusted comparisons were made against baseline, and missing values were handled without imputation.

Given the exploratory nature of the study and the limited sample size, correlation analyses were interpreted cautiously. The responder subgroup analysis based on short-term BCVA improvement was also exploratory and was performed to assess whether clinically meaningful visual gain was accompanied by distinct OCTA changes. This was a retrospective convenience sample; therefore, no *a priori* power calculation was performed.

## 3 Results

### 3.1 Baseline characteristics

A total of 20 eyes with nAMD and 20 cataract control eyes were included. The nAMD cohort included 11 eyes with typical nAMD, 4 eyes with polypoidal choroidal vasculopathy, and 5 eyes with retinal angiomatous proliferation. The two groups were comparable in age, sex distribution, and study-eye laterality, whereas baseline BCVA was worse in the nAMD group (*P* = 0.003), consistent with the clinical characteristics of the disease cohort. The baseline characteristics are summarized in Table 1.

**TABLE 1:** Baseline characteristics.

Characteristic	nAMD (*n* = 20)	Cataract (*n* = 20)	*P*-value
Age, years	75.00 ± 7.48	73.25 ± 7.48	0.464
Male sex, *n* (%)	12 (60.0%)	10 (50.0%)	0.751
Right eye, *n* (%)	8 (40.0%)	9 (45.0%)	1.000
logMAR BCVA	0.70 (0.40, 1.24)	0.46 (0.28, 0.60)	0.003*

Values are presented as mean ± standard deviation or median (IQR), as appropriate.

*P < 0.05.

### 3.2 Baseline aqueous humor cytokine profile in nAMD and cataract controls

At baseline, the aqueous humor profile in nAMD showed alterations in several angiogenesis- and inflammation-related mediators compared with cataract controls. Compared with cataract controls, eyes with nAMD showed significant differences in Ang-2 (*P* = 0.020), HGF (*P* = 0.002), VEGF-C (*P* = 0.001), IP-10 (*P* = 0.001), HB-EGF (*P* = 0.001), IL-8 (*P* = 0.048), and PlGF (*P* = 0.015). These data are summarized in Table 2.

**TABLE 2:** Baseline aqueous humor cytokines.

Biomarker	Cataract control (*n* = 20)	nAMD (*n* = 20)	*P*-value
Ang-2	31.93 (13.81, 42.85)	34.11 (31.93, 40.62)	0.020*
HGF	147.00 (97.84, 175.90)	214.94 (182.21, 267.54)	0.002*
PDGF-AA	23.18 (19.23, 28.37)	20.52 (17.00, 24.25)	0.130
VEGF-C	9.68 (9.68, 59.33)	90.15 (36.86, 110.57)	0.001*
IP-10	11.29 (6.02, 12.90)	18.57 (14.16, 26.91)	0.001*
bFGF	4.92 (2.42, 10.29)	3.34 (2.05, 6.25)	0.317
HB-EGF	1.71 (0.88, 3.55)	4.50 (3.23, 6.94)	0.001*
IL-8	4.17 (3.44, 5.48)	5.88 (3.86, 14.02)	0.048*
PlGF	2.50 (1.80, 3.07)	3.44 (2.71, 4.33)	0.015*

Values are presented as median (IQR). Cytokine concentrations are shown as pg/mL.

*P < 0.05.

### 3.3 Longitudinal OCTA and visual changes after aflibercept loading treatment

Longitudinal OCTA analysis showed that the vascular response was region-specific and became more evident by month 2. No OCTA parameter showed a statistically significant change from baseline to month 1 after adjustment. By month 2, significant decreases from baseline were observed in superficial parafoveal vessel density (adjusted *P* = 0.016), superficial perifoveal vessel density (adjusted *P* = 0.031), and deep perifoveal vessel density (adjusted *P* = 0.0009), whereas other OCTA parameters remained stable over time. BCVA also improved significantly after treatment, with median logMAR BCVA improving from 0.70 (0.55, 1.24) at baseline to 0.46 (0.30, 1.00) at month 2 (*P* = 0.001). The baseline-to-month 1 and baseline-to-month 2 mixed-effects results are summarized in Table 3.

**TABLE 3:** Longitudinal OCTA vessel density changes during aflibercept loading.

OCTA parameter	Baseline mean	Month 1 mean	Mean diff. (baseline - month 1)	95% CI	Adj. P	Month 2 mean	Mean diff. (baseline - month 2)	95% CI	Adj. P
Superficial whole VD	44.41	44.85	−0.4400	−3.138 to 2.258	0.896	42.26	2.145	−1.497 to 5.787	0.293
Superficial foveal VD	18.24	18.91	−0.6650	−8.032 to 6.702	0.967	18.81	−0.5700	−9.345 to 8.205	0.983
Superficial parafoveal VD	45.64	42.91	2.735	−0.4180 to 5.888	0.093	41.15	4.490	0.8174 to 8.163	0.016
Superficial perifoveal VD	47.09	45.69	1.407	−2.072 to 4.886	0.525	42.14	4.950	0.4462 to 9.454	0.031
Deep whole VD	43.17	41.95	1.220	−2.230 to 4.670	0.615	41.07	2.095	−0.7844 to 4.974	0.171
Deep foveal VD	34.16	29.80	4.355	−4.069 to 12.78	0.378	34.35	−0.1950	−8.332 to 7.942	0.998
Deep parafoveal VD	48.35	45.88	2.475	−0.6678 to 5.618	0.132	46.11	2.245	−1.124 to 5.614	0.219
Deep perifoveal VD	44.74	42.36	2.376	−1.932 to 6.685	0.321	39.64	5.100	2.368 to 7.832	0.0009

VD, vessel density; CI, confidence interval. Values are shown as model-estimated means. Mean difference was calculated as baseline minus follow-up; positive values indicate a decrease from baseline. Adjusted P values are from Dunnett-adjusted *post hoc* comparisons against baseline after mixed-effects modeling.

**FIGURE 1:**
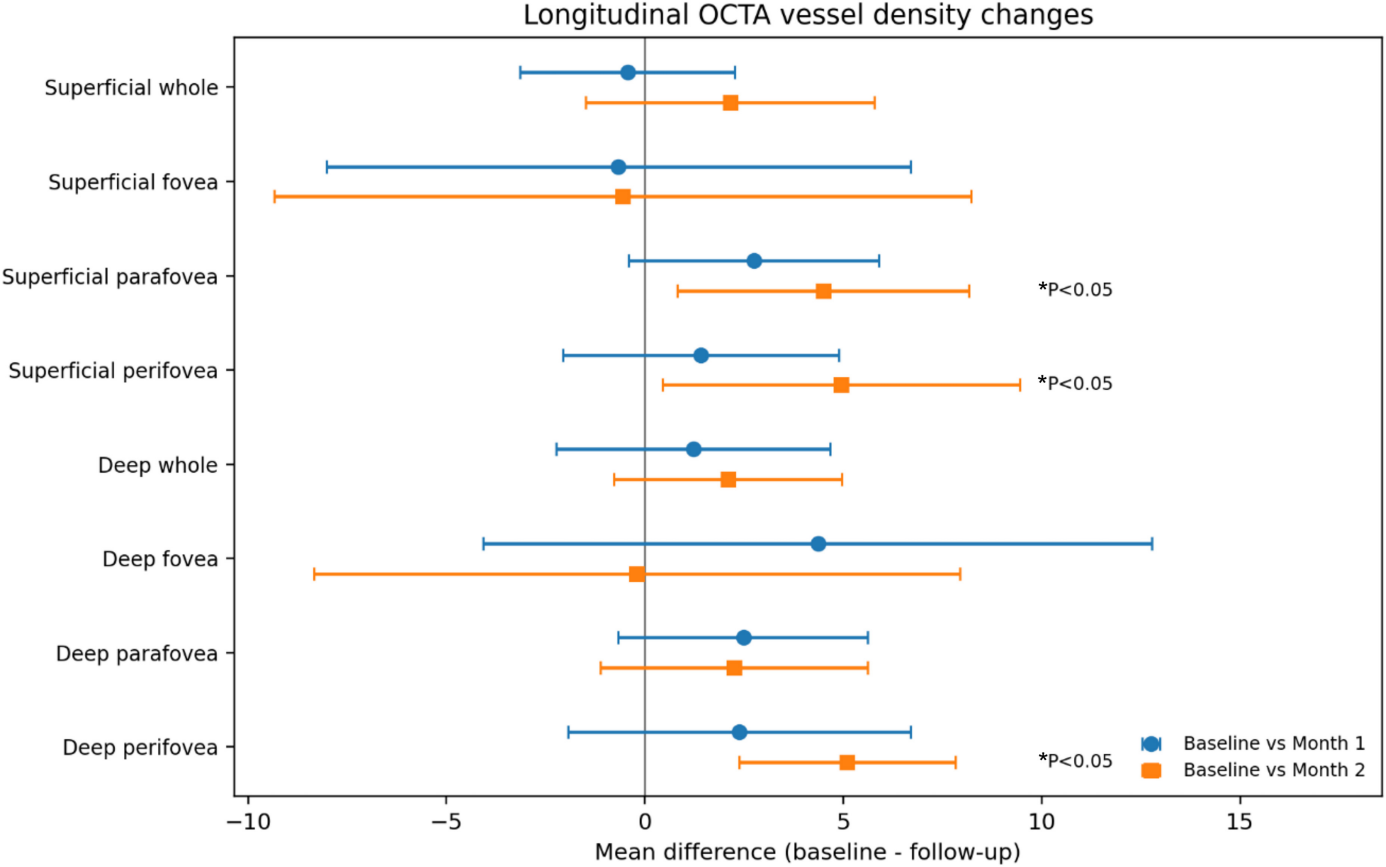
Longitudinal OCTA vessel density changes during aflibercept loading. Positive values indicate higher baseline vessel density than follow-up values, corresponding to reduced vessel density after treatment. *P < 0.05 versus baseline.

**FIGURE 2:**
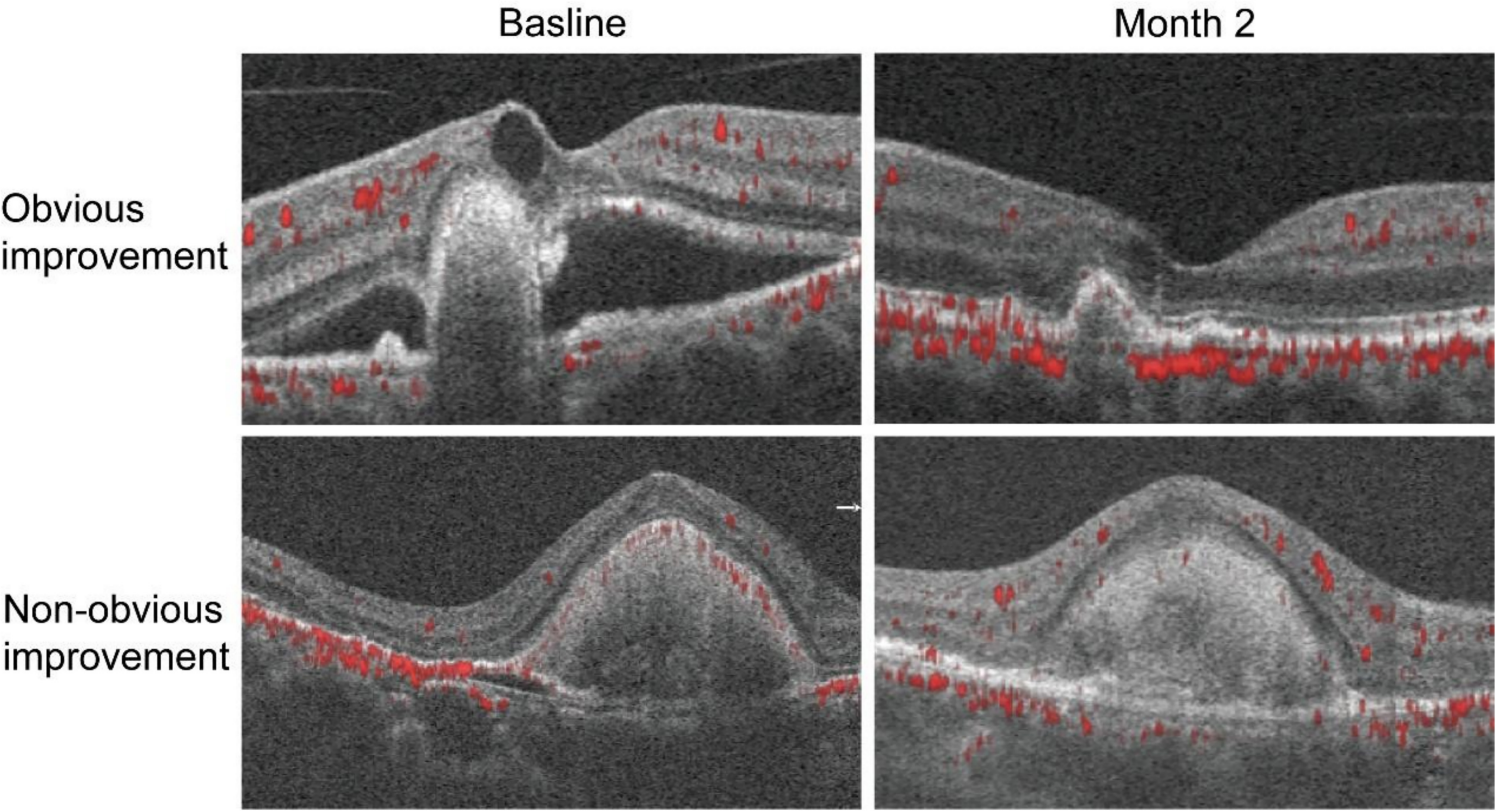
Representative OCTA B-scan images from an eye with obvious short-term visual improvement (upper row) and an eye with non-obvious short-term visual improvement (lower row) at baseline and month 2 during aflibercept loading.

Continuous correlation analyses did not demonstrate a consistent linear association between BCVA change and OCTA change across regions. For exploratory subgroup analysis, eyes were further classified according to whether short-term BCVA improvement reached at least 0.1 logMAR, a threshold chosen to reflect clinically meaningful visual gain during the loading phase. In the responder analysis, only the change in deep perifoveal vessel density differed significantly between the obvious-improvement and non-obvious-improvement groups. The responder finding was directionally consistent in sensitivity analyses using 0.05 and 0.15 logMAR thresholds (Supplementary Table 1). To further explore whether this signal was related to anatomical restoration, an additional SRF-based analysis was performed for deep perifoveal vessel density. Among the 18 eyes with available month 2 data, the SRF-resolved group showed a significantly greater decrease in deep perifoveal vessel density than the non-resolved group [median Delta VD, −7.25 vs. 3.10; exact Mann-Whitney U test, *P* = 0.0046]. Longitudinal OCTA changes are shown in Figure 1, representative OCTA B-scan images are shown in Figure 2, longitudinal OCTA model results are summarized in Table 3, and BCVA-OCTA correlations are summarized in Table 4.

**TABLE 4:** BCVA-OCTA correlations.

Analysis	Spearman r	*P*-value
Baseline BCVA vs. Δsuperficial parafovea	0.023	0.924
Baseline BCVA vs. Δsuperficial perifovea	−0.067	0.793
Baseline BCVA vs. Δdeep perifovea	0.020	0.938
ΔBCVA vs. Δsuperficial parafovea	−0.136	0.567
ΔBCVA vs. Δsuperficial perifovea	0.087	0.732
ΔBCVA vs. Δdeep perifovea	0.388	0.111
Baseline deep perifovea vs. ΔBCVA	−0.174	0.489
Baseline Ang-2 vs. ΔBCVA	−0.238	0.312
Baseline bFGF vs. ΔBCVA	0.131	0.583
Baseline IL-8 vs. ΔBCVA	−0.052	0.829

Δ Indicates change from baseline to month 2. Spearman r and unadjusted *P*-values are shown. BCVA, best-corrected visual acuity; OCTA, optical coherence tomography angiography.

### 3.4 Changes in aqueous humor cytokines

**FIGURE 3:**
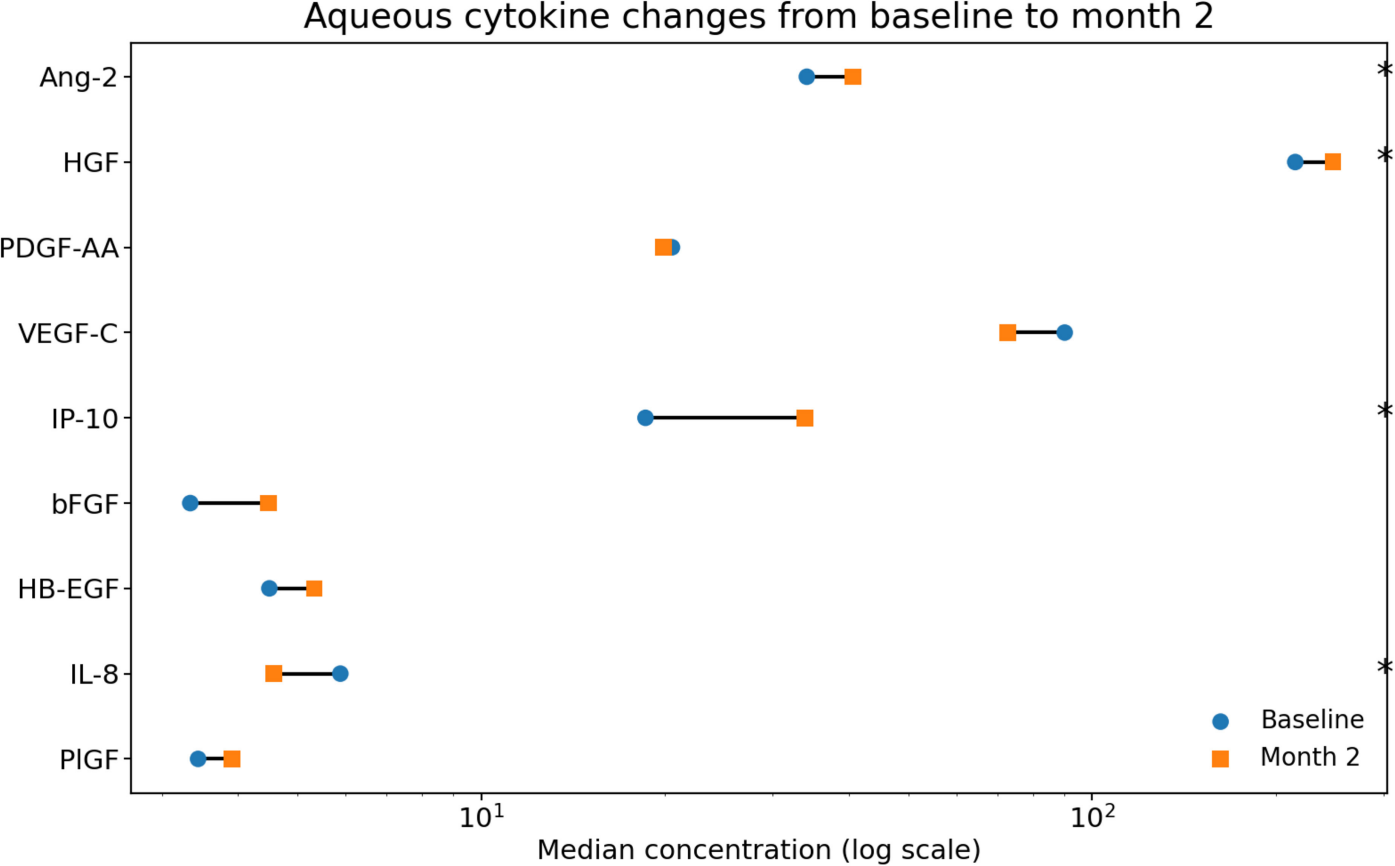
Aqueous cytokine changes from baseline to month 2 during aflibercept loading. Ang-2, HGF, and IP-10 increased significantly, whereas IL-8 decreased significantly after treatment. *P < 0.05.

Paired analyses indicated selective remodeling of the intraocular cytokine milieu after the loading phase of aflibercept. Ang-2 (*P* = 0.009), HGF (*P* = 0.002), and IP-10 (*P* < 0.001) increased, whereas IL-8 (*P* = 0.002) decreased. No statistically significant change was detected for PDGF-AA, VEGF-C, bFGF, HB-EGF, or PlGF. These findings suggest that aflibercept loading was accompanied by selective modulation of inflammatory and angiogenesis-related mediators within the intraocular milieu. These changes are illustrated in Figure 3.

### 3.5 Associations between aqueous humor biomarkers and OCTA remodeling

**FIGURE 4:**
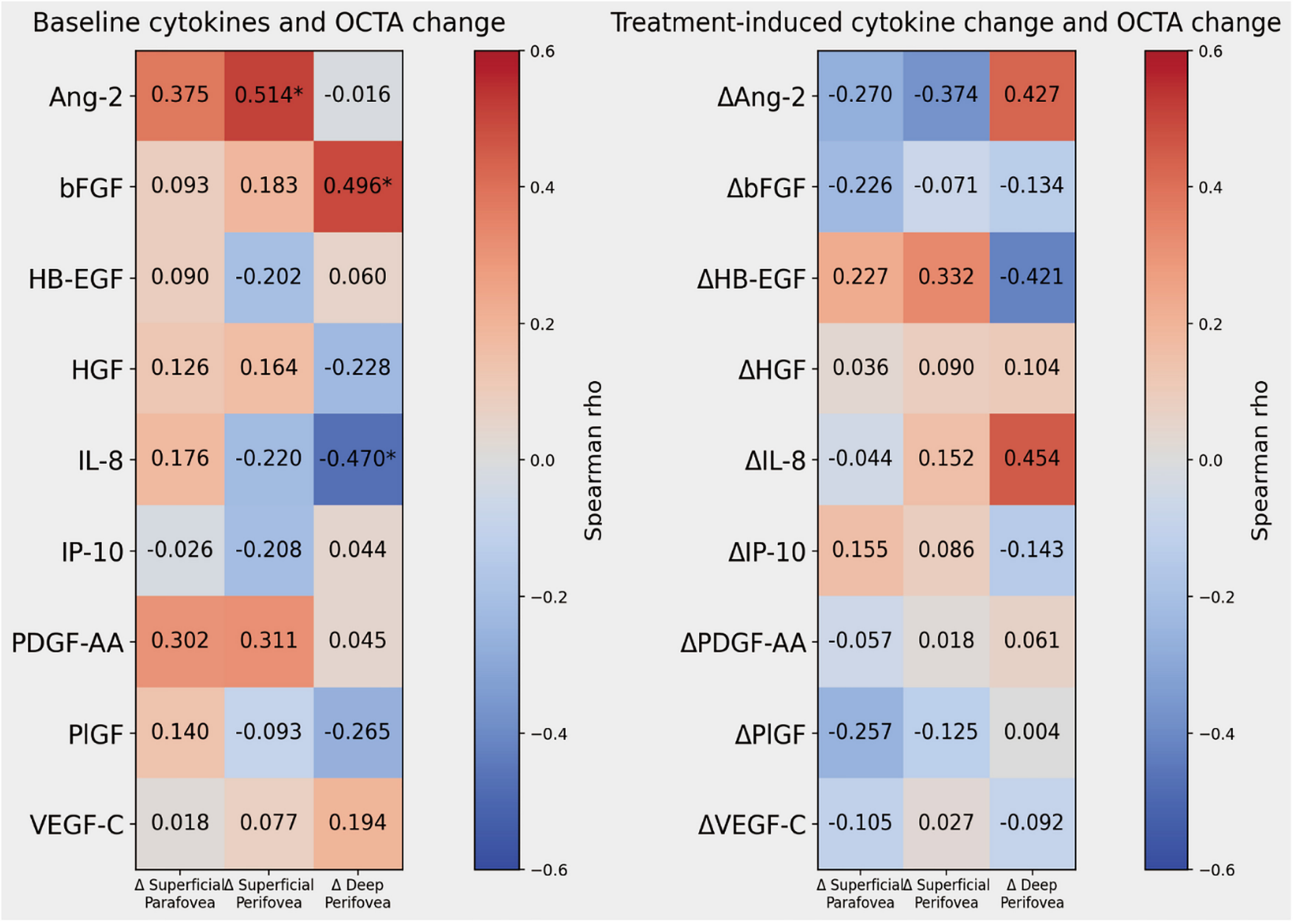
Correlations between aqueous humor biomarkers and OCTA changes. The left panel shows correlations of baseline cytokine levels with percent changes in vessel density at month 2, and the right panel shows correlations of treatment-induced cytokine changes with percent changes in vessel density at month 2. Nominal unadjusted associations are marked; FDR-adjusted results are provided in Supplementary Table 2. *Indicates a nominal unadjusted *P*-value < 0.05. None of these associations remained statistically significant after BH-FDR correction.

Exploratory correlation analyses suggested nominal associations between selected baseline cytokines and subsequent OCTA remodeling. However, after BH-FDR correction, the previously highlighted associations of baseline Ang-2, bFGF, and IL-8 with OCTA changes were no longer statistically significant. These results are therefore presented as hypothesis-generating signals, with full raw P values and q values shown in Supplementary Table 2. These correlations are summarized in Figure 4.

## 4 Discussion

In this exploratory study, we evaluated aqueous humor cytokines, layer-specific OCTA vessel density, and short-term visual outcomes during aflibercept loading in treatment-naive nAMD. Three main findings emerged. First, compared with cataract controls, eyes with nAMD showed a baseline cytokine profile that reflected both angiogenic and inflammatory activation beyond VEGF alone. Second, the cytokine response to aflibercept was selective rather than uniform. IL-8 decreased after treatment, whereas Ang-2, HGF, and IP-10 increased. Third, OCTA remodeling was regional and layer-specific, and the deep perifoveal region showed the most consistent signal associated with short-term visual improvement. Taken together, these findings suggest that early treatment response in nAMD is biologically heterogeneous and may involve non-VEGF pathway activity, although this remains indirect because VEGF-A was not measured.

At baseline, eyes with nAMD showed higher levels of Ang-2, HGF, VEGF-C, IP-10, HB-EGF, IL-8, and PlGF. These findings suggest that the intraocular microenvironment of nAMD is not driven solely by the VEGF-A pathway, but more likely reflects the concurrent involvement of vascular destabilization, chronic inflammatory activation, and potentially VEGF-independent pro-angiogenic signaling (4, 5, 23, 24). Among these alterations, the increase in Ang-2 is particularly noteworthy. As a key regulator of vascular stability, Ang-2 weakens Tie2-dependent homeostatic signaling and promotes endothelial activation, vascular leakage, and amplification of inflammation. Ng reported that aqueous humor levels of Ang-2, HGF, and IL-8 were all higher in nAMD eyes than in cataract controls, and that higher Ang-2 was associated with worse visual acuity and more severe macular edema, in line with the present findings (23). HGF and HB-EGF may more strongly reflect tissue repair activity and alternative pro-angiogenic signaling beyond VEGF (25, 26). The increases in IP-10 and IL-8 further indicate that the inflammatory network in nAMD may have a dual and complex nature (27, 28). These observations, together with the baseline increases in PlGF and VEGF-C, further support the concept that non-VEGF signaling may be involved before treatment initiation (29, 30).

The most clinically relevant finding of the present study is that cytokine remodeling during aflibercept loading was asynchronous. Rather than showing a uniform decline in disease-related mediators, the intraocular milieu shifted selectively after treatment. In particular, Ang-2 and HGF increased further after aflibercept loading. This finding should be interpreted in relation to the timing of aqueous humor sampling. Because the second sample was obtained immediately before the third injection, it corresponded to the end of the monthly dosing interval, and a trough-phase rebound-like increase in these mediators cannot be excluded. However, Ang-2 and HGF are not direct targets of aflibercept, and their increase is unlikely to indicate a simple loss of VEGF blockade. Instead, this pattern may reflect a reactive or counterregulatory remodeling response of VEGF-independent pathways after rapid VEGF-A suppression. This interpretation is consistent with previous biomarker studies. Angermann reported that aflibercept treatment was followed by an increase in Ang-2 and suggested that this may represent a potential escape mechanism (31). Cabral also showed that, despite VEGF-A suppression after bevacizumab, several other angiogenic biomarkers increased after treatment, including Ang-2, HGF, VEGF-C, and HB-EGF (32). Mantel further found that HGF and PAI-1 were associated with incomplete anti-VEGF response, suggesting that VEGF-independent pathways may contribute to persistent exudation and refractory disease (33). Our results are broadly consistent with these reports, but also add several new points. Similar to previous work, we found that Ang-2 and HGF were already elevated at baseline in treatment-naive nAMD eyes. More importantly, both markers increased further during aflibercept loading. Therefore, the observed increases may reflect compensatory non-VEGF pathway remodeling occurring near the trough phase of aflibercept exposure, rather than direct evidence of treatment failure or a confirmed causal escape mechanism. The increase in HGF may reflect activation of a VEGF-independent remodeling pathway. HGF/c-Met signaling can promote angiogenesis and tissue repair through mechanisms that are not directly blocked by aflibercept. Mantel et al. reported higher aqueous HGF levels in nAMD eyes with incomplete anti-VEGF response, and suggested that HGF-related pathways may contribute to residual exudative activity (33). Cabral et al. also found that HGF increased after anti-VEGF treatment, together with several other angiogenic mediators, despite VEGF-A suppression (32). These findings support the view that HGF may represent a compensatory non-VEGF pathway during anti-VEGF therapy. IP-10 should be interpreted differently. Previous studies showed that aqueous IP-10 is elevated in nAMD eyes and may increase further after aflibercept treatment (14, 16). However, IP-10 has context-dependent functions. It can participate in Th1-related inflammatory signaling, but it has also been described as an angiostatic or antiangiogenic mediator (16). Therefore, the post-treatment increase in IP-10 in the present study is more likely to reflect inflammatory and immune remodeling of the intraocular microenvironment, rather than a primary pro-angiogenic escape mechanism.

From a clinical standpoint, these findings suggest that Ang-2- and HGF-related compensation may be one of the key reasons why some eyes do not respond fully to anti-VEGF monotherapy. In nAMD, incomplete anti-VEGF response is commonly seen as persistent fluid, recurrent exudation or hemorrhage, progressive fibrosis, or limited visual recovery (34, 35). In this setting, our findings provide a biologically plausible explanation for why some eyes continue to show disease activity despite VEGF suppression. The lesion may not enter a true quiescent state. Instead, VEGF-dependent activity may be partly suppressed while other pathways remain active and continue to support leakage, instability, or recurrence. Recent faricimab studies provide indirect support for this view. In treatment-naive nAMD, faricimab reduced aqueous Ang-2, and higher baseline Ang-2 was associated with better treatment response (21). In eyes switched from aflibercept to faricimab, Ang-2 also decreased after switching, together with improvement in exudative changes (22). These findings suggest that Ang-2 is not only a marker of disease activity but also a clinically relevant pathway in eyes with persistent fluid or suboptimal response under VEGF-centered therapy. Our results extend this framework by suggesting that HGF may be another complementary escape pathway that deserves further study.

The OCTA results complement this molecular pattern. Post-treatment changes were not diffuse but were mainly observed in the parafoveal and perifoveal regions, especially in the deep perifoveal layer. This suggests that early vascular remodeling after aflibercept loading is localized rather than global. The deep perifoveal region may therefore be a relatively sensitive area for detecting early treatment-related change. Consistently, previous OCTA studies have suggested that treatment response, recurrent exudation, and neovascular activity are associated with specific lesion architecture and flow-related features rather than any single global density parameter (19, 36, 37). Importantly, the observed decrease in vessel density should not be interpreted too simplistically as impaired perfusion. In the setting of nAMD treatment, reduced OCTA vessel density may also reflect regression of abnormal flow signals, vascular remodeling after exudation control, or changes in segmentation and projection conditions as retinal anatomy stabilizes. Accordingly, the deep perifoveal signal identified here may be better understood as a marker of treatment-related microvascular remodeling than as direct evidence of worsening ischemia. The additional SRF-based analysis further supports this interpretation. Eyes with SRF resolution showed a significantly greater decrease in deep perifoveal vessel density than eyes with persistent SRF. This association suggests that the observed “density loss” may partly reflect anatomical restoration after exudation control, regression of abnormal exudation-related flow signals, or reduced fluid-related projection and segmentation interference. Nevertheless, OCTA-derived vessel density cannot fully distinguish physiological DCP perfusion from lesion-related or artifact-related signals. Therefore, potential suppression of the normal deep capillary plexus cannot be completely excluded. This interpretation is also consistent with the structure of our functional results. Continuous-variable analyses did not demonstrate a stable linear association between OCTA change and BCVA improvement across regions, whereas exploratory subgroup analysis suggested that the change in deep perifoveal vessel density was the only OCTA parameter that differed significantly between eyes with and without clinically meaningful short-term visual gain. This pattern suggests that the relationship between microvascular change and vision in nAMD is likely nonlinear and influenced by multiple additional factors, including lesion location, exudative burden, outer retinal integrity, and irreversible tissue damage. Therefore, deep perifoveal remodeling should be regarded as an exploratory but potentially informative signal, rather than as a validated surrogate for visual outcome. Accordingly, reduced OCTA vessel density should be interpreted cautiously as an exploratory remodeling signal rather than as a validated surrogate of favorable visual outcome.

Further exploratory analyses suggested that baseline cytokine status may be modestly associated with subsequent OCTA remodeling, particularly in the perifoveal region. Because these nominal associations did not survive BH-FDR correction, they should be interpreted as hypothesis-generating only. In the present dataset, baseline cytokines were not directly associated with BCVA improvement, and treatment-induced cytokine changes were not significantly associated with OCTA change. Therefore, the associations of Ang-2, bFGF, and IL-8 with OCTA change require validation in larger cohorts with longer follow-up.

This study has several limitations. The sample size was small, and the retrospective exploratory design limits statistical robustness and generalizability. This was a convenience sample without an *a priori* power calculation. The correlation and subgroup analyses were exploratory, and nominal cytokine-OCTA associations did not survive FDR correction. Follow-up was limited to the loading phase, so we cannot determine whether the cytokine and OCTA changes identified here predict longer-term anatomical control or visual prognosis. Moreover, VEGF-A was not included in the final cytokine analysis, which precluded calculation of the Ang-2/VEGF-A ratio as an index of vascular stability and prevented direct confirmation of VEGF-A suppression. Subtype-stratified descriptive data are provided in Supplementary Table 3, but the small subtype-specific sample sizes precluded meaningful stratified testing. In addition, aqueous humor does not fully represent the molecular environment of the macular lesion, and cataract eyes, while practical controls, do not provide a true physiological reference. Finally, although an additional SRF-based analysis was performed, other structural OCT biomarkers were not comprehensively quantified, and their independent effects on visual outcome could not be assessed.

## 5 Conclusion

In conclusion, the early response to aflibercept in treatment-naive nAMD appears to be characterized not by uniform molecular suppression, but by selective cytokine remodeling accompanied by regional OCTA change. The post-treatment increase in Ang-2 and HGF is particularly noteworthy and is consistent with possible activation of VEGF-independent pathways under VEGF suppression. At the imaging level, the deep perifoveal region showed the most consistent association with short-term visual improvement, suggesting that this area may be especially sensitive to early treatment-related microvascular remodeling. Although these findings remain exploratory, they support the integration of aqueous humor biomarkers, structural OCT findings, and regional OCTA metrics for future response stratification in nAMD. Larger prospective studies with longer follow-up are needed to validate this framework. AI-based quantitative imaging analysis may further improve the objectivity and reproducibility of treatment-response assessment.

## Data Availability

The raw data supporting the conclusions of this article will be made available by the authors, without undue reservation.

## References

[1] Guymer RH Campbell TG. Age-related macular degeneration. *Lancet.* (2023) 401:1459–72. 10.1016/S0140-6736(22)02609-5 36996856

[2] Reitan G Kjellevold Haugen IB Andersen K Bragadottir R Bindesbøll C. Through the eyes of patients: understanding treatment burden of intravitreal anti-VEGF injections for nAMD patients in Norway. *Clin Ophthalmol*. (2023) 17:1465–74. 10.2147/OPTH.S409103 37256195 PMC10226541

[3] Rosenberg D Deonarain DM Gould J Sothivannan A Phillips MR Sarohia GS et al. Efficacy, safety, and treatment burden of treat-and-extend versus alternative anti-VEGF regimens for nAMD: a systematic review and meta-analysis. *Eye*. (2023) 37:6–16. 10.1038/s41433-022-02020-7 35396574 PMC9829919

[4] Dervenis N Dervenis P Agorogiannis E. Neovascular age-related macular degeneration: disease pathogenesis and current state of molecular biomarkers predicting treatment response-a scoping review. *BMJ Open Ophthalmol*. (2024) 9:e001516. 10.1136/bmjophth-2023-001516 38341189 PMC10862285

[5] Joo JH Kim H Shin JH Moon SW. Aqueous humor cytokine levels through microarray analysis and a sub-analysis based on optical coherence tomography in wet age-related macular degeneration patients. *BMC Ophthalmol*. (2021) 21:399. 10.1186/s12886-021-02152-6 34794403 PMC8603589

[6] Cao X Sanchez JC Dinabandhu A Guo C Patel TP Yang Z et al. Aqueous proteins help predict the response of patients with neovascular age-related macular degeneration to anti-VEGF therapy. *J Clin Invest*. (2022) 132:e144469. 10.1172/JCI144469 34874918 PMC8759792

[7] Heier JS Singh RP Wykoff CC Csaky KG Lai TYY Loewenstein A et al. THE ANGIOPOIETIN/TIE PATHWAY IN RETINAL VASCULAR DISEASES: a review. *Retina*. (2021) 41:1–19. 10.1097/IAE.0000000000003003 33136975

[8] Akwii RG Sajib MS Zahra FT Mikelis CM. Role of angiopoietin-2 in vascular physiology and pathophysiology. *Cells*. (2019) 8:471. 10.3390/cells8050471 31108880 PMC6562915

[9] Joussen AM Ricci F Paris LP Korn C Quezada-Ruiz C Zarbin M. Angiopoietin/Tie2 signalling and its role in retinal and choroidal vascular diseases: a review of preclinical data. *Eye*. (2021) 35:1305–16. 10.1038/s41433-020-01377-x 33564135 PMC8182896

[10] Heier JS Khanani AM Quezada Ruiz C Basu K Ferrone PJ Brittain C et al. Efficacy, durability, and safety of intravitreal faricimab up to every 16 weeks for neovascular age-related macular degeneration (TENAYA and LUCERNE): two randomised, double-masked, phase 3, non-inferiority trials. *Lancet*. (2022) 399:729–40. 10.1016/S0140-6736(22)00010-1 35085502

[11] Agostini H Abreu F Baumal CR Chang DS Csaky K G Demetriades AM et al. Faricimab for neovascular age-related macular degeneration and diabetic macular edema: from preclinical studies to phase 3 outcomes. *Graefes Arch Clin Exp Ophthalmol*. (2024) 262:3437–51. 10.1007/s00417-024-06531-9 38847896 PMC11584429

[12] Mao J Chen N Zhang S Fang Y Zheng Z Wu S et al. Association between inflammatory cytokines in the aqueous humor and hyperreflective foci on optical coherence tomography in patients with neovascular age-related macular degeneration and polypoidal choroidal vasculopathy. *Front Med*. (2022) 9:973025. 10.3389/fmed.2022.973025 36213652 PMC9538653

[13] Arai Y Takahashi H Inoda S Tan X Sakamoto S Inoue Y et al. Aqueous humour proteins and treatment outcomes of anti-VEGF therapy in neovascular age-related macular degeneration. *PLoS One*. (2020) 15:e0229342. 10.1371/journal.pone.0229342 32155173 PMC7064238

[14] Sato T Takeuchi M Karasawa Y Takayama K Enoki T. Comprehensive expression patterns of inflammatory cytokines in aqueous humor of patients with neovascular age-related macular degeneration. *Sci Rep*. (2019) 9:19447. 10.1038/s41598-019-55191-x 31857597 PMC6923359

[15] Connolly E El-Farouki G Brennan K Cahill M Doyle SL. Poor response to bevacizumab correlates with higher IL-6 and IL-8 aqueous cytokines in AMD. *Invest Ophthalmol Vis Sci*. (2024) 65:37. 10.1167/iovs.65.11.37 39325472 PMC11437685

[16] Sato T Takeuchi M Karasawa Y Enoki T Ito M. Intraocular inflammatory cytokines in patients with neovascular age-related macular degeneration before and after initiation of intravitreal injection of anti-VEGF inhibitor. *Sci Rep*. (2018) 8:1098. 10.1038/s41598-018-19594-6 29348424 PMC5773499

[17] Faatz H Lommatzsch A. Overview of the use of optical coherence tomography angiography in neovascular age-related macular degeneration. *J Clin Med*. (2024) 13:5042. 10.3390/jcm13175042 39274255 PMC11396513

[18] Rispoli M Cennamo G Antonio LD Lupidi M Parravano M Pellegrini M et al. Practical guidance for imaging biomarkers in exudative age-related macular degeneration. *Surv Ophthalmol*. (2023) 68:615–27. 10.1016/j.survophthal.2023.02.004 36854371

[19] Hsu CR Lai TT Hsieh YT Ho TC Yang CM Yang CH. Combined quantitative and qualitative optical coherence tomography angiography biomarkers for predicting active neovascular age-related macular degeneration. *Sci Rep*. (2021) 11:18068. 10.1038/s41598-021-97652-2 34508170 PMC8433312

[20] Pu J Zhuang X Li M Zhang X Su Y He G et al. Analyzing formation and absorption of avascular subretinal hyperreflective material in nAMD From OCTA-based insights. *Am J Ophthalmol*. (2024) 267:192–203. 10.1016/j.ajo.2024.06.019 38914153

[21] Nonogaki R Ota H Takeuchi J Nakano Y Sajiki AF Todoroki T et al. Analysis of the aqueous humor before and after the administration of faricimab in patients with nAMD. *Sci Rep*. (2024) 14:31951. 10.1038/s41598-024-83473-6 39738534 PMC11685434

[22] Todoroki T Takeuchi J Ota H Nakano Y Sajiki AF Nakamura K et al. Aqueous humor cytokine analysis in age-related macular degeneration after switching from aflibercept to faricimab. *Invest Ophthalmol Vis Sci*. (2024) 65:15. 10.1167/iovs.65.11.15 39250120 PMC11385661

[23] Ng DS Yip YW Bakthavatsalam M Chen LJ Ng TK Lai TY et al. Elevated angiopoietin 2 in aqueous of patients with neovascular age related macular degeneration correlates with disease severity at presentation. *Sci Rep*. (2017) 7:45081. 10.1038/srep45081 28345626 PMC5366858

[24] Cao Y Dang M Tian Z Zhang T Hou L Wang M et al. Aqueous humor cytokine levels in patients with subretinal fibrosis in neovascular age-related macular degeneration. *BMC Ophthalmol*. (2024) 24:335. 10.1186/s12886-024-03614-3 39129024 PMC11318135

[25] Inoue Y Shimazawa M Nakamura S Takata S Hashimoto Y Izawa H et al. Both autocrine signaling and paracrine signaling of HB-EGF enhance ocular neovascularization. *Arterioscler Thromb Vasc Biol*. (2018) 38:174–85. 10.1161/ATVBAHA.117.310337 29191924

[26] Chen YJ Tsai RK Wu WC He MS Kao YH Wu WS. Enhanced PKCδ and ERK signaling mediate cell migration of retinal pigment epithelial cells synergistically induced by HGF and EGF. *PLoS One*. (2012) 7:e44937. 10.1371/journal.pone.0044937 23028692 PMC3447816

[27] Bodnar RJ Yates CC Wells A. IP-10 blocks vascular endothelial growth factor-induced endothelial cell motility and tube formation via inhibition of calpain. *Circ Res*. (2006) 98:617–25. 10.1161/01.RES.0000209968.66606.10 16484616 PMC3826264

[28] Chen L Zhang H Zhang Y Li X Wang M Shen Y et al. Ganglion cell-derived LysoPS induces retinal neovascularisation by activating the microglial GPR34-PI3K-AKT-NINJ1 axis. *J Neuroinflammation*. (2024) 21:278. 10.1186/s12974-024-03265-7 39468551 PMC11520652

[29] Leitch IM Gerometta M Eichenbaum D Finger RP Steinle NC Baldwin ME. Vascular endothelial growth factor C and D signaling pathways as potential targets for the treatment of neovascular age-related macular degeneration: a narrative review. *Ophthalmol Ther*. (2024) 13:1857–75. 10.1007/s40123-024-00973-4 38824253 PMC11178757

[30] Motohashi R Noma H Yasuda K Kotake O Goto H Shimura M. Dynamics of soluble vascular endothelial growth factor receptors and their ligands in aqueous humour during ranibizumab for age-related macular degeneration. *J Inflamm*. (2018) 15:26. 10.1186/s12950-018-0203-x 30534004 PMC6280338

[31] Angermann R Rauchegger T Nowosielski Y Seifarth C Egger S Kralinger MT et al. Systemic counterregulatory response of angiopoietin-2 after aflibercept therapy for nAMD: a potential escape mechanism. *Acta Ophthalmol*. (2021) 99:e869–75. 10.1111/aos.14691 33326179 PMC8519089

[32] Cabral T Lima LH Mello LGM Polido J Correa ÉP Oshima A et al. Bevacizumab injection in patients with neovascular age-related macular degeneration increases angiogenic biomarkers. *Ophthalmol Retina*. (2018) 2:31–7. 10.1016/j.oret.2017.04.004 29376143 PMC5783314

[33] Mantel I Borgo A Guidotti J Forestier E Kirsch O Derradji Y et al. Molecular biomarkers of neovascular age-related macular degeneration with incomplete response to anti-vascular endothelial growth factor treatment. *Front Pharmacol*. (2020) 11:594087. 10.3389/fphar.2020.594087 33447243 PMC7802772

[34] Mettu PS Allingham MJ Cousins SW. Incomplete response to Anti-VEGF therapy in neovascular AMD: exploring disease mechanisms and therapeutic opportunities. *Prog Retin Eye Res*. (2021) 82:100906. 10.1016/j.preteyeres.2020.100906 33022379 PMC10368393

[35] Sepah YJ Do DV Mesquida M Day BM Blotner S Afridi R et al. Aqueous humour interleukin-6 and vision outcomes with anti-vascular endothelial growth factor therapy. *Eye*. (2024) 38:1755–61. 10.1038/s41433-024-03015-2 38622330 PMC11156666

[36] Jia H Lu B Zhao Z Yu Y Wang F Zhou M et al. Prediction of the short-term efficacy of anti-VEGF therapy for neovascular age-related macular degeneration using optical coherence tomography angiography. *Eye Vis*. (2022) 9:16. 10.1186/s40662-022-00287-1 35505390 PMC9066856

[37] Faatz H Rothaus K Ziegler M Book M Spital G Lange C et al. The architecture of macular neovascularizations predicts treatment responses to anti-VEGF therapy in neovascular AMD. *Diagnostics*. (2022) 12:2807. 10.3390/diagnostics12112807 36428867 PMC9688972

