# Supplementary material for: Dynamic changes in aqueous humor cytokines and OCTA parameters during aflibercept loading in treatment-naive neovascular age-related macular degeneration
Source: Front Med (Lausanne). 2026 Jul 30;13:1857826. doi: 10.3389/fmed.2026.1857826 (PMC13470260; doi:10.3389/fmed.2026.1857826)
Supplement: Supplementary file 1 [file Table_1.pdf]

Supplementary Tables

Supplementary Table S1. Sensitivity analyses using alternative BCVA improvement thresholds.

BCVA improvement threshold	Metric	Improved n	Improved median (IQR)	Non-improved n	Non-improved median (IQR)	Mann-Whitney U	P value
Delta BCVA <= -0.05 logMAR	Delta superficial parafoveal VD	14	-6.60 (-13.65, 3.33)	6	-17.35 (-21.23, -6.35)	62.0	0.1093
Delta BCVA <= -0.05 logMAR	Delta superficial perifoveal VD	13	-7.80 (-23.20, 4.40)	5	2.10 (-6.70, 3.60)	25.0	0.5028
Delta BCVA <= -0.05 logMAR	Delta deep perifoveal VD	13	-15.70 (-18.50, -7.80)	5	1.10 (-5.50, 11.20)	11.0	0.0350
Delta BCVA <= -0.10 logMAR	Delta superficial parafoveal VD	12	-6.60 (-12.75, 0.97)	8	-16.80 (-20.07, -2.70)	65.0	0.2083
Delta BCVA <= -0.10 logMAR	Delta superficial perifoveal VD	11	-7.80 (-17.15, 2.20)	7	2.10 (-7.90, 4.00)	33.0	0.6590
Delta BCVA <= -0.10 logMAR	Delta deep perifoveal VD	11	-17.10 (-18.90, -8.30)	7	1.10 (-6.90, 13.50)	10.0	0.0083
Delta BCVA <= -0.15 logMAR	Delta superficial parafoveal VD	11	-5.00 (-13.20, 2.15)	9	-15.20 (-19.50, -3.40)	66.0	0.2299
Delta BCVA <= -0.15 logMAR	Delta superficial perifoveal VD	10	-7.20 (-20.17, 3.95)	8	-2.30 (-8.12, 3.80)	36.0	0.7618
Delta BCVA <= -0.15 logMAR	Delta deep perifoveal VD	10	-16.90 (-19.10, -8.05)	8	-2.20 (-9.20, 12.35)	15.0	0.0266

Notes: Delta values indicate changes from baseline to month 2. The primary threshold was Delta BCVA <= -0.10 logMAR; alternative thresholds were -0.05 and -0.15 logMAR.
