# Supplementary material for: Dynamic changes in aqueous humor cytokines and OCTA parameters during aflibercept loading in treatment-naive neovascular age-related macular degeneration
Source: Front Med (Lausanne). 2026 Jul 30;13:1857826. doi: 10.3389/fmed.2026.1857826 (PMC13470260; doi:10.3389/fmed.2026.1857826)
Supplement: Supplementary file 2 [file Table_2.pdf]

Supplementary Table S2. Spearman correlations with BH-FDR correction.

family	analyte	comparison	Spearman r	95% CI	n	raw P	BH-FDR q
baseline cytokines	Ang2-pre	Ang2_pre vs. pct_dSparafovea_2m_pre	0.3748	-0.09515 to 0.7081	20	0.1035	0.7762
baseline cytokines	Ang2-pre	Ang2_pre vs. pct_dSperifovea_2m_pre	0.5140	0.04703 to 0.7966	18	0.0291	0.4930
baseline cytokines	Ang2-pre	Ang2_pre vs. pct_dDperifovea_2m_pre	-0.0159	-0.4907 to 0.4661	18	0.9501	0.9501
cytokine changes	dAng2	dAng2 vs. pct_dSparafovea_2m_pre	-0.2699	-0.6447 to 0.2095	20	0.2497	0.9870
cytokine changes	dAng2	dAng2 vs. pct_dSperifovea_2m_pre	-0.3736	-0.7228 to 0.1278	18	0.1268	0.9510
cytokine changes	dAng2	dAng2 vs. pct_dDperifovea_2m_pre	0.4272	-0.06444 to 0.7520	18	0.0770	0.8200
cytokine changes	dFGF2	dFGF2 vs. pct_dSparafovea_2m_pre	-0.2264	-0.6168 to 0.2534	20	0.3371	0.9870
cytokine changes	dFGF2	dFGF2 vs. pct_dSperifovea_2m_pre	-0.0712	-0.5316 to 0.4216	18	0.7788	0.9870
cytokine changes	dFGF2	dFGF2 vs. pct_dDperifovea_2m_pre	-0.1342	-0.5757 to 0.3679	18	0.5954	0.9870
cytokine changes	dHB_EGF	dHB_EGF vs. pct_dSparafovea_2m_pre	0.2274	-0.2524 to 0.6174	20	0.3349	0.9870
cytokine changes	dHB_EGF	dHB_EGF vs. pct_dSperifovea_2m_pre	0.3320	-0.1742 to 0.6994	18	0.1784	0.9870
cytokine changes	dHB_EGF	dHB_EGF vs. pct_dDperifovea_2m_pre	-0.4209	-0.7486 to 0.07212	18	0.0820	0.8200
cytokine changes	dHGF	dHGF vs. pct_dSparafovea_2m_pre	0.0361	-0.4246 to 0.4819	20	0.8799	0.9870
cytokine changes	dHGF	dHGF vs. pct_dSperifovea_2m_pre	0.0898	-0.4062 to 0.5449	18	0.7231	0.9870
cytokine changes	dHGF	dHGF vs. pct_dDperifovea_2m_pre	0.1042	-0.3939 to 0.5550	18	0.6806	0.9870
cytokine changes	dIL8	dIL8 vs. pct_dSparafovea_2m_pre	-0.0436	-0.4877 to 0.4184	20	0.8551	0.9870
cytokine changes	dIL8	dIL8 vs. pct_dSperifovea_2m_pre	0.1518	-0.3523 to 0.5876	18	0.5477	0.9870
cytokine changes	dIL8	dIL8 vs. pct_dDperifovea_2m_pre	0.4543	-0.03089 to 0.7662	18	0.0582	0.8200
cytokine changes	dIP10	dIP10 vs. pct_dSparafovea_2m_pre	0.1549	-0.3215 to 0.5687	20	0.5144	0.9870
cytokine changes	dIP10	dIP10 vs. pct_dSperifovea_2m_pre	0.0857	-0.4096 to 0.5419	18	0.7354	0.9870
cytokine changes	dIP10	dIP10 vs. pct_dDperifovea_2m_pre	-0.1434	-0.5820 to 0.3597	18	0.5701	0.9870
cytokine changes	dPDGF_AA	dPDGF_AA vs. pct_dSparafovea_2m_pre	-0.0571	-0.4980 to 0.4072	20	0.8109	0.9870
cytokine changes	dPDGF_AA	dPDGF_AA vs. pct_dSperifovea_2m_pre	0.0175	-0.4648 to 0.4919	18	0.9449	0.9870

cytokine changes	dPDGF_AA	dPDGF_AA vs. pct_dDperifovea_2m_pre	0.0609	-0.4301 to 0.5241	18	0.8103	0.9870
cytokine changes	dPIGF	dPlGF vs. pct_dSparafovea_2m_pre	-0.2574	-0.6368 to 0.2223	20	0.2732	0.9870
cytokine changes	dPIGF	dPlGF vs. pct_dSperifovea_2m_pre	-0.1251	-0.5695 to 0.3759	18	0.6210	0.9870
cytokine changes	dPIGF	dPlGF vs. pct_dDperifovea_2m_pre	0.0041	-0.4753 to 0.4817	18	0.9870	0.9870
cytokine changes	dVEGF	dVEGF vs. pct_dSparafovea_2m_pre	0.0962	-0.3738 to 0.5270	20	0.6865	0.9870
cytokine changes	dVEGF	dVEGF vs. pct_dSperifovea_2m_pre	-0.1373	-0.5778 to 0.3652	18	0.5871	0.9870
cytokine changes	dVEGF	dVEGF vs. pct_dDperifovea_2m_pre	0.0052	-0.4745 to 0.4825	18	0.9838	0.9870
cytokine changes	dVEGF_C	dVEGF_C vs. pct_dSparafovea_2m_pre	-0.1046	-0.5330 to 0.3666	20	0.6609	0.9870
cytokine changes	dVEGF_C	dVEGF_C vs. pct_dSperifovea_2m_pre	0.0268	-0.4575 to 0.4989	18	0.9158	0.9870
cytokine changes	dVEGF_C	dVEGF_C vs. pct_dDperifovea_2m_pre	-0.0919	-0.5464 to 0.4044	18	0.7169	0.9870
baseline cytokines	FGF2-pre	FGF2_pre vs. pct_dSparafovea_2m_pre	0.0933	-0.3764 to 0.5248	20	0.6957	0.9501
baseline cytokines	FGF2-pre	FGF2_pre vs. pct_dSperifovea_2m_pre	0.1828	-0.3241 to 0.6081	18	0.4679	0.9358
baseline cytokines	FGF2-pre	FGF2_pre vs. pct_dDperifovea_2m_pre	0.4956	0.02245 to 0.7874	18	0.0365	0.4930
baseline cytokines	HB_EGF-pre	HB_EGF_pre vs. pct_dSparafovea_2m_pre	0.0901	-0.3791 to 0.5225	20	0.7055	0.9501
baseline cytokines	HB_EGF-pre	HB_EGF_pre vs. pct_dSperifovea_2m_pre	-0.2020	-0.6205 to 0.3061	18	0.4215	0.9358
baseline cytokines	HB_EGF-pre	HB_EGF_pre vs. pct_dDperifovea_2m_pre	0.0604	-0.4305 to 0.5237	18	0.8119	0.9501
baseline cytokines	HGF-pre	HGF_pre vs. pct_dSparafovea_2m_pre	0.1263	-0.3473 to 0.5486	20	0.5957	0.9406
baseline cytokines	HGF-pre	HGF_pre vs. pct_dSperifovea_2m_pre	0.1641	-0.3412 to 0.5958	18	0.5153	0.9406
baseline cytokines	HGF-pre	HGF_pre vs. pct_dDperifovea_2m_pre	-0.2281	-0.6370 to 0.2811	18	0.3627	0.9358
baseline cytokines	IL8-pre	IL8_pre vs. pct_dSparafovea_2m_pre	0.1759	-0.3019 to 0.5831	20	0.4581	0.9358
baseline cytokines	IL8-pre	IL8_pre vs. pct_dSperifovea_2m_pre	-0.2198	-0.6318 to 0.2891	18	0.3808	0.9358
baseline cytokines	IL8-pre	IL8_pre vs. pct_dDperifovea_2m_pre	-0.4696	-0.7741 to 0.01152	18	0.0493	0.4930
baseline cytokines	IP10-pre	IP10_pre vs. pct_dSparafovea_2m_pre	-0.0256	-0.4738 to 0.4332	20	0.9147	0.9501
baseline cytokines	IP10-pre	IP10_pre vs. pct_dSperifovea_2m_pre	-0.2079	-0.6243 to 0.3005	18	0.4079	0.9358
baseline cytokines	IP10-pre	IP10_pre vs. pct_dDperifovea_2m_pre	0.0445	-0.4435 to 0.5121	18	0.8609	0.9501

baseline cytokines	PDGF_AA-pre	PDGF_AA_pre vs. pct_dSparafovea_2m_pre	0.3024	-0.1755 to 0.6649	20	0.1951	0.8974
baseline cytokines	PDGF_AA-pre	PDGF_AA_pre vs. pct_dSperifovea_2m_pre	0.3108	-0.1970 to 0.6871	18	0.2094	0.8974
baseline cytokines	PDGF_AA-pre	PDGF_AA_pre vs. pct_dDperifovea_2m_pre	0.0454	-0.4427 to 0.5128	18	0.8579	0.9501
baseline cytokines	PIGF-pre	PlGF_pre vs. pct_dSparafovea_2m_pre	0.1402	-0.3348 to 0.5584	20	0.5554	0.9406
baseline cytokines	PIGF-pre	PlGF_pre vs. pct_dSperifovea_2m_pre	-0.0932	-0.5473 to 0.4033	18	0.7131	0.9501
baseline cytokines	PIGF-pre	PlGF_pre vs. pct_dDperifovea_2m_pre	-0.2650	-0.6598 to 0.2445	18	0.2879	0.9358
baseline cytokines	VEGF-pre	VEGF_pre vs. pct_dSparafovea_2m_pre	-0.3167	-0.6736 to 0.1601	20	0.1737	0.8974
baseline cytokines	VEGF-pre	VEGF_pre vs. pct_dSperifovea_2m_pre	-0.1394	-0.5792 to 0.3633	18	0.5812	0.9406
baseline cytokines	VEGF-pre	VEGF_pre vs. pct_dDperifovea_2m_pre	-0.0475	-0.5143 to 0.4410	18	0.8515	0.9501
baseline cytokines	VEGF_C-pre	VEGF_C_pre vs. pct_dSparafovea_2m_pre	0.0183	-0.4391 to 0.4682	20	0.9389	0.9501
baseline cytokines	VEGF_C-pre	VEGF_C_pre vs. pct_dSperifovea_2m_pre	0.0769	-0.4170 to 0.5356	18	0.7618	0.9501
baseline cytokines	VEGF_C-pre	VEGF_C_pre vs. pct_dDperifovea_2m_pre	0.1937	-0.3139 to 0.6152	18	0.4412	0.9358

Notes: Raw P values and Benjamini-Hochberg false discovery rate (BH-FDR) q values are shown for cytokine-OCTA correlation analyses.
