# Supplementary material for: Dynamic changes in aqueous humor cytokines and OCTA parameters during aflibercept loading in treatment-naive neovascular age-related macular degeneration
Source: Front Med (Lausanne). 2026 Jul 30;13:1857826. doi: 10.3389/fmed.2026.1857826 (PMC13470260; doi:10.3389/fmed.2026.1857826)
Supplement: Supplementary file 3 [file Table_3.pdf]

Supplementary Table S3. Baseline characteristics stratified by nAMD subtype.

Variable	typical nAMD (n=11)	PCV (n=4)	RAP (n=5)
n	11	4	5
Age	78.00 (71.00, 82.00)	73.00 (68.75, 77.25)	71.00 (68.00, 79.00)
BCVA_pre_logMAR	0.82 (0.70, 1.30)	0.50 (0.28, 0.87)	0.60 (0.40, 1.05)
Delta_BCVA_logMAR	-0.18 (-0.25, -0.04)	-0.11 (-0.19, -0.02)	-0.22 (-0.38, 0.00)
Ang-2	31.93 (27.48, 53.88)	35.81 (29.70, 48.99)	40.62 (31.93, 40.62)
HGF	213.10 (176.78, 244.44)	197.67 (168.53, 220.47)	236.19 (219.21, 293.07)
PDGF-AA	20.89 (16.82, 27.30)	20.60 (19.85, 21.55)	17.00 (17.00, 20.68)
VEGF-C	110.57 (72.79, 110.57)	56.66 (32.49, 82.55)	86.25 (27.12, 122.06)
IP-10	22.25 (17.30, 31.84)	14.87 (11.29, 16.91)	19.45 (14.23, 20.68)
bFGF	3.34 (2.33, 11.00)	2.58 (1.77, 3.75)	3.69 (2.00, 4.38)
HB-EGF	4.50 (3.87, 7.87)	5.12 (3.75, 6.03)	3.23 (3.23, 5.73)
IL-8	6.80 (3.80, 12.12)	4.82 (3.86, 10.31)	5.31 (4.21, 22.87)
PlGF	3.86 (3.02, 4.38)	2.60 (2.04, 2.81)	3.65 (3.65, 4.49)
VEGF	20.55 (12.82, 62.02)	41.47 (26.27, 52.71)	43.44 (25.26, 60.00)
Deep whole VD	44.60 (41.50, 45.25)	41.05 (34.20, 48.10)	42.70 (41.50, 43.20)
Deep foveal VD	35.30 (26.35, 45.15)	31.70 (29.42, 34.58)	34.60 (27.20, 37.30)
Deep parafoveal VD	50.40 (45.20, 51.75)	42.95 (37.83, 50.22)	48.60 (44.40, 50.20)
Deep perifoveal VD	45.70 (45.20, 46.60)	42.95 (40.73, 45.17)	42.45 (40.65, 42.85)
Superficial whole VD	46.80 (43.65, 48.10)	45.70 (43.53, 47.33)	39.80 (38.50, 41.50)
Superficial foveal VD	22.90 (10.65, 24.60)	18.35 (12.65, 26.80)	14.90 (11.70, 15.70)
Superficial parafoveal VD	47.40 (45.20, 50.35)	45.80 (42.85, 48.62)	39.50 (38.10, 47.30)
Superficial perifoveal VD	47.50 (46.55, 48.80)	50.70 (46.67, 55.70)	40.65 (38.83, 41.80)

Notes: Values are presented as median (IQR) unless otherwise indicated. Data are descriptive only; no between-subtype hypothesis testing was performed.
